# Exosomes in the Repair of Bone Defects: Next-Generation Therapeutic Tools for the Treatment of Nonunion

**DOI:** 10.1155/2019/1983131

**Published:** 2019-08-04

**Authors:** Jian Lu, Qi-Yang Wang, Jia-Gen Sheng

**Affiliations:** ^1^Department of Orthopaedic Surgery, Shanghai Jiao Tong University Affiliated Sixth People's Hospital, 600 Yishan Road, Shanghai 200233, China; ^2^The Third Affiliated Hospital of Soochow University, Changzhou, Jiangsu 213003, China

## Abstract

Nonunion with bone defects, a common complication after long bone fracture, is a major challenge for orthopaedic surgeons worldwide because of the high incidence rate and difficulties in achieving successful treatment. Bone defects are the main complications of nonunion. The conventional biological treatments for nonunion with bone defects involve the use of autologous bone grafts or bone graft substitutes and cell-based therapy. Traditional nonunion treatments have always been associated with safety issues and various other complications. Bone grafts have limited autologous cancellous bone and there is a risk of infection. Additionally, problems with bone graft substitutes, including rejection and stimulation of bone formation, have been noted, and the health of the stem cell niche is a major consideration in cell-based therapy. In recent years, researchers have found that exosomes can be used to deliver functional RNA and mediate cell-to-cell communication, suggesting that exosomes may repair bone defects by regulating cells and cytokines involved in bone metabolism. In this review, we highlight the possible relationships between risk factors for nonunion and exosomes. Additionally, we discuss the roles of exosomes in bone metabolism and bone regeneration.

## 1. Introduction

Traumatic and life-threatening fractures of the long bones have increased dramatically as the demand for motor transport continues to increase in developing countries [1]. Nonunion is the most common complication after long bone fracture, and the rate of nonunion is estimated to be between 5% and 10% [2, 3]. In addition, the higher incidence rates of obesity and musculoskeletal diseases and reduced rates of bone regeneration [4] have increased the occurrence of nonunion [5]. Nonunion is not limited to developing countries but is experienced worldwide. According to the Food and Drug Administration, nonunion is considered if the fracture is not healed after a minimum of 9 months and there are no obvious progressive signs of healing for 3 consecutive months [6]. Patients with bone nonunion often require two or more surgeries, resulting in severe psychological and economic pressure [7, 8] and seriously affecting quality of life [5, 9]. Therefore, an in-depth understanding of the fracture healing process and related mechanisms and the provision of appropriate interventions to accelerate bone regeneration are essential to avoid adverse consequences. Thus, given the burden of nonunion on patients and society, new treatments for nonunion intervention are urgently needed.

**Figure 1:**
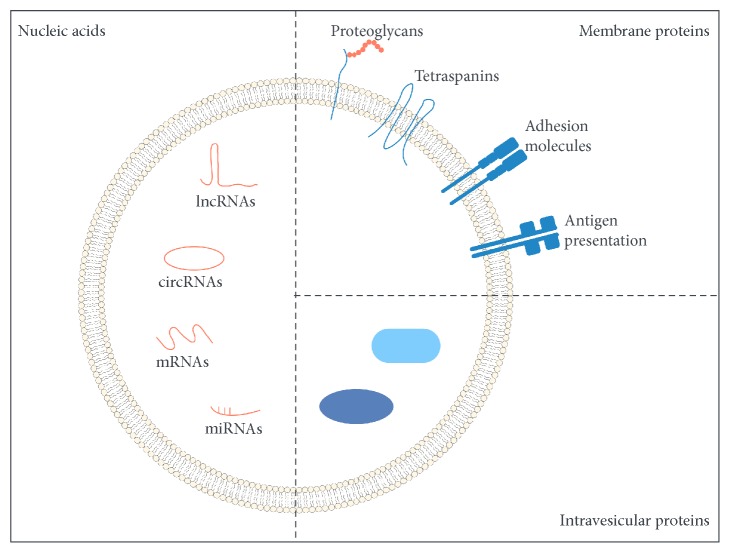
The constitute of exosomes.

Extracellular vesicles are a class of heterogeneous membrane vesicles including three major subpopulations (exosomes, microvesicles, and apoptotic bodies), which has a great diversity of biophysical properties and functions. The International Society for Extracellular Vesicles updated their guidelines in 2018 (Table 1) [10]. Exosomes contain lipids, nucleic acids, proteins, and signalling molecules (Figure 1) [11]. Exosomes were first discovered and described as exfoliated membrane vesicles in 1981 [12]. Firstly, endosomes are produced by plasma membrane internalization of donor cells, and then the proteins and RNAs (including lncRNAs, circRNAs, mRNAs, and miRNAs) are selectively crated into the multivesicular bodies, via endosomal sorting complex required for transport- (ESCRT-) dependent or ESCRT-independent mechanisms. Subsequently, the multivesicular bodies are either fused to the lysosome for degradation or released into the extracellular space by fusion with the plasma membrane to produce exosomes. Microvesicles are produced directly on the plasma membrane {Tao, 2018 #264}. Today, exosomes are defined as cellular organelles that are released by various tissues and cells and can be internalized by receptor cells through endocytosis [13]. Exosomes are widely distributed and can be separated from almost all types of biological fluids, particularly semen [14], breast milk [15], saliva [16], synovial fluid [17], and urine [18]. Recent studies have demonstrated that exosomes possess the ability to stimulate the regeneration and repair of tissue and organs [19], including the heart [20], skin [21], and liver [22]. In the past two decades, exosomes have been shown to have various applications as naturally derived nanoparticles. Endocytosis of exosomes can facilitate the absorption of proteins, mRNAs, and microRNAs, thereby affecting target cells [23]. Moreover, these functional RNAs enable intercellular signal communication through exosomes [24].

In this review, we discuss the applications of exosome treatment in nonunion with bone defects, highlighting the physiological mechanisms of fracture, risk factors that may cause nonunion, classifications of nonunion, and treatment intervention using exosomes.

## 2. Fracture Healing

**Figure 2:**
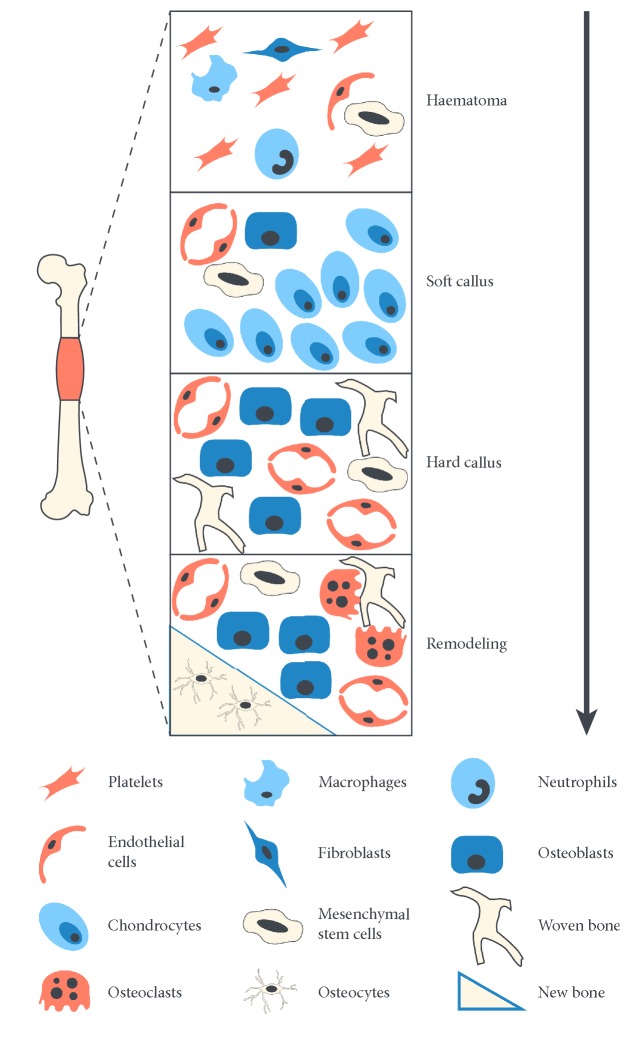
Cells involved in the process of bone healing.

Bone can heal without scarring and return to its original state, unlike other tissues [25], through the concerted activities of thousands of genes, cytokines, growth factors, chemokines, and other molecules [26]. The newly healed bone is similar in structure and mechanical properties to the original bone [27–29]. Fracture healing involves an initial anabolic stage in which tissue volume increases in relation to newly generated and differentiated stem cells, which form bone and vascular tissue (Figure 2) [30]. First, the injured bone forms a hematoma, which is a fibrin clot caused by periosteal blood vessel haemorrhage beneath the periosteum and the medullary canal [31] as a result of coagulation [32]. Although the mechanical properties are poor, the fibrin clot provides the first step for fracture connection [33]. Simultaneously, macrophages, degranulating platelets, and other inflammatory cells [34] reach the fracture site to clean up the wound [35]. Gradually, endothelial cells and fibroblasts infiltrate, forming new capillaries and collagen matrix, which results in the formation of granulation tissue [32, 36] to fill the fracture gap [37]. Initial granulation tissue is then gradually replaced by fibrous tissue, to form a soft callus [38]. Close to the hypoxic fracture line, as chondrocytes become hypertrophic and begin to undergo apoptosis, the soft callus exhibits endochondral ossification [26]. In the periphery of the new cartilage tissue toward the fracture location, periosteum swelling and bone formation occur through intramembranous ossification [39]. Additionally, following exposure to vascular endothelial growth factor secreted from endothelial cells, the surrounding matrix is digested by chondrocytes and then infiltrated by blood vessels and osteoblasts [40]. The next phase involves the formation of the primary bone [34], which is characterized by replacement of mineralized bone and high levels of osteoblast activity [41], concurrent with cartilage tissue development.

### 2.1. Indirect (Secondary) Fracture Healing

Indirect healing with callus formation is an orderly process of bone recombination [42]. In general, using fixation with steel plates and screws, the fracture ends are better restored and compressively fixed. Micromotion and weight-bearing enhance indirect fracture healing. However, delayed union and nonunion can also appear when there is too much motion [43].

### 2.2. Direct Fracture Healing

Direct fracture healing can occur by lamellar bone remodelling when the fracture end is very stable with no gap formation. However, when natural healing of the fracture does not occur, this type of healing usually requires open reduction and internal fixation [42].

## 3. Contribution of Risk factors to Fracture Nonunion

The relationships between various risk factors affecting fracture healing and nonunion have been confirmed in recent studies [44]. Risk factors identified as contributing to the development of fracture nonunion include patient-dependent factors, such as age [45] (age-related changes can affect many biological processes during fracture healing), sex [46] (high oestrogen levels in postmenopausal women play important roles in promoting bone formation), nutritional state [47, 48] (during the bone regeneration process, there is an increase in metabolic requirements), diabetes [49] (diabetes is a metabolic disorder that interferes with bone formation and damages fracture healing), osteoporosis [50] (osteoporosis impairs bone regeneration and the ability to restore biomechanical properties), alcohol abuse [51] (alcohol reduces bone repair, repaired tissue stiffness, and ash density), smoking [52] (nicotine inhibits osteoblast proliferation and is a vasoconstrictor, leading to tissue ischemia and hypoxia), and nonsteroidal anti-inflammatories (NSAIDs) [53] (long-term, high-dose treatment with NSAIDs reduces osteoblast numbers and inhibits the formation of prostaglandin). Patient-independent factors include fracture gap [54] (the fracture process is poor when the fracture gap is greater than 2 cm), fracture site [55] (compared with bone fracture of the diaphysis, the incidence of healing defects in the metaphyseal fracture is lower and the healing time is shorter), type of fracture [55, 56] (compared with stable and simple fractures, the more ruptured and unstable the fracture ends, the higher the risk of debris ischemia and necrosis), and infection [57, 58] (infection reduces the strength of the callus and creates conditions for sequestrum and osteolysis). All strategies that help shorten healing time and restore work and activity faster not only improve patient outcomes but also help reduce the financial burden of fracture and nonunion.

## 4. Classification of Nonunion

The most common classification of nonunions is the Weber and Cech classification [59]. Nonunions include hypertrophic nonunion and atrophic nonunion. Hypertrophic nonunion, also called mechanical nonunion, involves a large number of nonbridged calluses containing cartilage and is characterized by excessive bone formation and poor mechanical fixation. Atrophic nonunion, also called biological nonunion, is characterized by minimal callus or cartilage owing to lack of blood supply or cells. In atrophic nonunion, the fracture end may be hardened or osteoporotic [60].

## 5. Treatment Intervention

Long bone nonunion is often accompanied with bone defects. For management of nonunion, the main strategies include removal of necrotic bone and tissue, filling most of the bone defect, promoting the recruitment of osteoblasts, increasing the concentrations of osteoinductive substances, and providing a stable mechanical environment [61]. The distribution of blood vessels at the site of nonunion has also been shown to be an important factor in fracture healing [62]. In this section, we summarize various types of reconstruction treatments to achieve bone healing and maintain limb length.

### 5.1. Surgical Intervention

In order to regenerate hard and soft tissue defects, mechanical stability can be promoted through surgical intervention [61].

*Nail dynamization*: After treatment of long bone fractures with intramedullary nails, dynamization of intramedullary nails may help increase axial compression and micromotion to stimulate healing [63, 64]. This method may cause axial shortening of the femur via dynamization [65].

*Exchange nailing with augmentation plating*: Exchange nailing provides biological effects by increasing subperiosteal blood circulation and stimulating osteogenesis [66], growth factor activation [67, 68], and inflammatory responses [69]. As the length of the medullary stenosis and diameter of the intramedullary nail increase, the effective contact area between the intramedullary nail and the medullary cavity is significantly increased, thereby enhancing mechanical stability [70, 71].

*Augmentation plating*: Rotational instability is also a risk factor in diaphyseal long bone nonunion, which can be solved by augmentation plating [72, 73].

*External fixation*: External fixation to treat nonunion provides tension and support at the long bone nonunion sites for bone binding [74, 75]. The procedure can improve early weight bearing while increasing the stability of bone healing [76]. The disadvantages of this procedure are that it takes a long time to heal and that the wound is at risk of infection [77].

However, surgical intervention can only overcome factors affecting instability and is not sufficient to cure nonunion. Treatment of nonunion involves not just providing a stable mechanical environment for nonunion but also increasing osteogenic activity to address biological nonunion.

### 5.2. Autograft and Other Bone Graft Substitutes

Autologous bone grafts are the “gold standard” for the treatment of nonunion because of complete histocompatibility and strong osteoconduction, osteoinduction, and osteogenic activities [78]. However, autograft bone grafts may result in increased blood loss, pain, and possible infection at the donor site [79]. Although allogeneic bone has no osteogenic potential, it can be used as a scaffold to provide osteoconductive material [80]. Demineralized bone matrix is composed of collagen, noncollagenous proteins, bone morphogenic proteins, and growth factors [81], conferring the bone with osteoinductive and some oeteoconductive properties [82]. Ceramics, such as calcium phosphate [83], tricalcium phosphate [84], hydroxyapatite [85], and calcium sulphate [86], have been widely used as osteoinductive carriers and transplant substitutes. Among them, calcium phosphate is chemically similar to human bone minerals and also have bioconductive [87]. Le Nihouannen D [88] proved that microporous biphasic calcium phosphate containing hydroxyapatite and beta-tricalcium phosphate implants into sheep muscle can promote bone formation. Le Nihouannen D et al. [89] also used calcium phosphate ceramics as scaffold combined with fibrin glue based composites to verify osteoinduction (new bone formation) and osteoconduction (bone healing capacity). However, allogeneic bone and bone graft substitutes may be associated with infection [90] and graft-versus-host disease [91]. Moreover, bone graft substitutes have no cellular components, and their effects are not as good as autologous bone.

### 5.3. Cell Therapy

Cell-based therapies, which apply a stem cell self-sufficient biological environment and heterogeneity to restore and improve tissue function, have been extensively investigated. Friendstein et al. found that, after hematopoietic necrosis, osteoblast-like bone marrow cells formed new bone* in vitro*, and mesenchymal stem cells (MSCs) were first isolated in this context [92]. Bruder et al. demonstrated for the first time that MSCs isolated from human bone marrow could regenerate normal bone in critical tibial defects of immunocompromised rats [93]. Additionally, MSCs were found to be ideal cells for bone tissue regeneration, not only because of their therapeutic potential and ability to self-renew but also because of their availability from many different tissues [94–96].

*Bone marrow-derived stem cells (BMSCs)*: BMSCs are the most abundant cells in the bone marrow. BMSCs play roles in regulating hematopoietic stem cells and progenitor cells through different signalling pathways, as demonstrated in various studies [97]. When treating nonunion, Connolly et al. reported that 18 of 20 patients with ununited tibial fractures were successfully cured by autologous marrow injection into the nonunion site [98]. Additionally, Quarto et al. [99] and Marcacci et al. [100] have achieved healing results with bone graft substitutes loaded with BMSCs. Similar treatments such as BMSCs and biphasic calcium phosphate biomaterials have been transplanted in nonunion to achieve effective fracture healing and bone growth in clinical trials [101]. This work was supported by European Union's Seventh Framework Programme (FP7/FP7-HEALTH-2009).

*Induced pluripotent stem cells (iPSCs)*: Human iPSCs are similar to embryonic stem cells in multilineage differentiation potential and proliferation ability [102]. Teramura et al. [103] demonstrated that mouse iPSCs could be induced into MSC-like cells and then differentiated into osteoblasts. Although the study of iPSCs is still relatively new, iPSCs may have promising applications in the healing of bone defects.

Other stem cells also have shown excellent osteogenesis capacity. For example, endothelial progenitor cells can form ectopic vascular bone for the treatment of critical size bone defects [104]. In animal studies hydroxyapatite-tricalcium phosphate containing allogeneic MCSs were effective in enhancing the repair of critical-sized defect in the canine femur [105, 106]. Although the treatment outcomes of these novel methods are promising, the molecular mechanisms of MSC repair* in vivo* are still unclear. Increasing evidence suggests that the therapeutic effects of MSCs are related to exosome-mediated paracrine induction [107, 108].

### 5.4. Cell-Free Therapy

**Figure 3:**
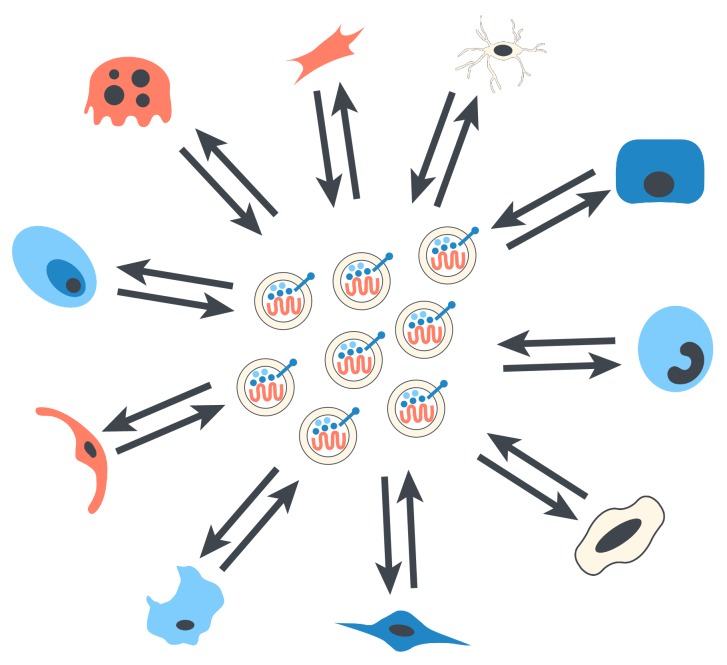
Exosomes are likely to be the way cells communicate with each other in the process of bone healing.

Many studies have supported the roles of exosomes in intercellular communication through paracrine signalling in various tissue repair processes and diseases (Figure 3) [109]. These natural mechanisms can be applied as intercellular signalling pathways to stimulate bone regeneration [109]. Therefore, cell-free therapies that increase the formation of osteoblasts and the interactions between cells may have potential applications in the treatment of nonunion.

As an important component of exosomes, microRNAs have attracted much attention in the study of exosome function owing to their important regulatory roles. Li et al. reported that osteoclast-derived exosomal* miR-214-3p* inhibits osteogenic activity and reduces bone formation; additionally, inhibition of* miR-214-3p* in osteoclasts may have applications in the treatment of nonunion [110]. Qin and colleagues found that muscle-secreting myostatin inhibits osteoblastic differentiation by blocking osteocyte-derived exosomal* miR-218*, suggesting the presence of a potential communication mechanism between muscle and bone [111]. Weilner et al. reported that* miR-31* from vascular endothelial cell-derived exosomes may be a biomarker and potential therapeutic target for osteoporosis [112].

Proteins in exosomes in cell-to-cell communication also play important roles. Ge et al. reported that highly expressed proteins in MC3T3 cell-derived exosomes are rich in osteogenic-related pathways [113]. Moreover, Huynh et al. reported that receptor activator of nuclear factor-*κ*B ligand (RANK), which is highly enriched in exosomes derived from osteoclasts, is a paracrine regulator of osteoclastogenesis [114].

Many recent studies have evaluated the application of stem cell-derived exosomes for bone repair. Qin et al. demonstrated that BMSC-derived exosomes regulate osteoblast expression by* miR-196a in vitro* and improve bone regeneration ability in a Sprague-Dawley rat model of calvarial defects [115]. Additionally, angiogenesis has been shown to be an essential factor in bone regeneration. Ashoo et al. demonstrated that BMSC-derived exosomes are capable of increasing endothelial cell viability* in vitro* and stimulating angiogenesis* in vivo* [116]. Lu et al. also reported that adipose stem cell-derived exosomes promote the proliferation and differentiation of human primary osteoblastic cells [117]. Exosomes released from adipose stem cells (ASCs) have also been shown to promote ASCs-induced angiogenesis [118].

Proteins and RNAs contained in osteoblasts-derived exosomes played an important role in intercellular communication within bone tissue. Ge et al. demonstrated that osteoblast-derived exosomes activate eukaryotic factor 2 to promote osteoblastic differentiation* in vitro* [113]. Weilner et al. demonstrated that galectin-3 levels in osteoblasts-derived exosomes were positively correlated with osteogenesis potential [119]. A proteomic study of exosomes derived from MC3T3 cells (mouse osteoblasts) revealed some osteogenesis-related pathways, including integrin signalling, mammalian target of rapamycin (mTOR) signalling, and eukaryotic initiation factor 2 (EIF2) signalling [113]. Moreover, Cui et al. reported that mineralizing osteoblast-derived exosomes promoted bone marrow stromal cell differentiation into osteoblasts by activating Wnt signalling [120].

Exosomes have been widely reported in the field of regeneration. Qi et al. found that exosomes secreted by MSCs derived from human induced pluripotent stem cells (hiPSC-MSC-Exos) could significantly promote osteogenesis and angiogenesis in rats with osteoporosis [121]. Liu et al. found that, in a model of steroid-induced osteonecrosis in rats, hiPSC‐MSC‐Exos could prevent femoral head necrosis by activating PI3K/Akt signaling pathways in endothelial cells to promote angiogenesis [122]. In another study, compared with exosomes secreted by synovial membrane MSCs (SMMSC-Exos), exosomes secreted by induced pluripotent stem cell-derived MSCs (iMSC-Exos) had superior therapeutic effect on osteoarthritis (OA) due to the ability to promote chondrocyte migration and proliferation [123]. Du et al. reported that hiPSC‐MSC‐Exos could alleviate hepatic ischemia-reperfusion (I/R) injury and promote cell proliferation in a rat model of hepatic I/R injury [124].

## 6. Discussion and Conclusion

Nonunion has major implications for patients and families and can also affect society. Surgical intervention can only solve mechanical nonunion, and interventions for biological bone nonhealing often involve autologous bone or bone graft substitutes, stem cell therapy, and other methods. Nonviable vesicles, such as exosomes, are associated with a lower risk of complications than cell-based treatment. Importantly, exosomes can be stored at −20°C for 6 months without loss of efficacy [125]. Thus, it is particularly important to grasp the relationship between exosome and nonunion healing mechanisms and to solve the problem of nonunion.

Osteoblasts, osteoclasts, proangiogenic factors, and blood platelets play important roles in fracture healing. As described above, osteoblasts release exosomes in a positive feedback loop to promote bone growth [113]. Inder and colleagues reported that prostate cancer cell-derived exosomes can attenuate osteoclast formation and stimulate osteoblast proliferation [126]. In addition to osteoclast-derived exosomes, which can affect osteoclast differentiation, Raimondi et al. reported that multiple myeloma-derived exosomes increase C-X-C motif chemokine receptor 4 expression and activation, thus promoting osteoclast maturation [127]. Additionally, Solberg et al. showed that lysosomal membrane protein 1-positive exosomes contain RANK ligand, osteoprotegerin, and tartrate-resistant acid phosphatase isolated from rat osteoblasts and osteocytes [128]. Osteoclast formation can be stimulated by RANKL-rich osteoblast-derived exosomes, as shown by Deng et al. [129]. In promoting angiogenesis, exosomes can stimulate endothelial cell migration and angiogenesis through exosomal* miR-129* and* miR-136* [116]. Torreggiani et al. reported that BMSCs treated with platelet-derived exosomes containing proteins and noncoding RNAs showed a significant increase in osteogenesis [130]. Understanding the processes and secreted components of cells involved in fracture healing may guide the development of new treatments for nonunion.

There are some factors that can explain the nonunion of fractures, and treatment of nonunion with these factors can be achieved through the use of exosomes. Xu et al. found that* miR-31a-5p* in rat bone marrow stromal cell-derived exosomes prevents age-related bone loss and reduces osteoclast activity in rats. Thus, they proposed that* miR-31a-5p* may be an age-related potential therapeutic target [131]. Notably, women have a higher probability of developing arthritis than men, and women who are postmenopausal are at increased risk [132]; Kolhe et al. noted that differences in miRNA expression were greater in women with osteoarthritis than in men with osteoarthritis [133], suggesting that some differences in miRNA contents may be related to sex. Additionally, Saha et al. analysed plasma-derived exosomes from alcoholic individuals and reported that alcohol increases exosome production in monocytes; they also found that exosomes containing* miR-27a* released from monocytes promoted naïve monocyte differentiation into M2 macrophages [134]. Goerzl et al. demonstrated that aspirin significantly reduces the levels of plasma platelet-derived exosomes without changing the total number of exosomes [135].

Exosomes have great potential for applications in the treatment of nonunion with bone defects and can be used to adjust the immune microenvironment and promote vascularization, proliferation, differentiation, and mineralization of osteoblasts. Exosomes are eliminated from the blood stream in short time and aggregate in the liver [136, 137]. Thus, future studies are needed to further assess the application and efficiency of exosome-based targeted drug delivery.

## Figures and Tables

**Table 1:** Types of extracellular vesicles.

Vesicles	Size (nm)	Origin
Exosomes	50 - 100	Endosomes
Microvesicles	100 – 1,000	Plasma membrane
Apoptotic bodies	1,000 – 5,000	Plasma membrane
